# XAF1 overexpression exacerbates diabetes by promoting pancreatic β-cell apoptosis

**DOI:** 10.1007/s00592-022-01930-y

**Published:** 2022-07-13

**Authors:** Yuki Nishimura, Misaki Iwashita, Masato Hayashi, Takanori Shinjo, Yukari Watanabe, Tatsuro Zeze, Akiko Yamashita, Takao Fukuda, Terukazu Sanui, Tomomi Sano, Tomoichiro Asano, Fusanori Nishimura

**Affiliations:** 1 Department of Periodontology, Division of Oral Rehabilitation, Faculty of Dental Science, Kyushu University, 3-1-1 Maidashi, Higashi-ku, Fukuoka, 812-8582 Japan; 2 Department of Cell Biology and Pharmacology, Faculty of Dental Science, Kyushu University, Fukuoka, Japan; 3 Department of Medical Chemistry, Division of Molecular Medical Science, Graduate School of Biomedical and Health Sciences, Hiroshima University, Hiroshima, Japan

**Keywords:** X-linked inhibitor of apoptosis–associated factor 1, Interferon β, β-cell apoptosis, High-fat diet

## Abstract

**Aims:**

Pancreatic β-cell apoptosis may be involved in the onset and progression of type 2 diabetes mellitus, although its mechanism remains unclear. We previously demonstrated that macrophage-derived interferon (IFN) β induced X-linked inhibitor of apoptosis–associated factor 1 (XAF1) expression in β-cells and accelerated β-cell apoptosis in vitro. Here, we explored the effects of XAF1 on β-cell function and progression of diabetes in vivo.

**Methods:**

Pancreatic β-cell-selective XAF1 overexpressing (*Xaf1* Tg) mice were generated. *Xaf1* Tg mice and their wild-type (WT) littermates were fed either a normal diet or a 40% or 60% high-fat diet (HFD). The effects of β-cell XAF1 on β-cell apoptosis and exacerbation of diabetes were investigated.

**Results:**

Palmitic acid induced IFNβ expression in macrophages, and HFD intake promoted macrophage infiltration in pancreatic islets, both of which cooperatively upregulated XAF1 expression in mouse islets. Furthermore, HFD-fed *Xaf1* Tg mice demonstrated increased β-cell apoptosis, lowered insulin expression, and impaired glucose tolerance compared with WT mice fed the same diet. These effects were more pronounced in the 60%HFD group than in the 40%HFD group.

**Conclusions:**

Pancreatic β-cell XAF1 expression was enhanced via HFD-induced, macrophage-derived IFNβ, which promoted β-cell apoptosis and led to a reduction in insulin secretion and progression of diabetes. To our knowledge, this is the first report to demonstrate an association between pancreatic β-cell XAF1 overexpression and exacerbation of diabetes, thus providing insight into the mechanism of β-cell mass reduction in diabetes.

**Supplementary Information:**

The online version contains supplementary material available at 10.1007/s00592-022-01930-y.

## Introduction

The onset and progress of type 2 diabetes mellitus are mainly caused by insulin resistance and pancreatic β-cell dysfunction. Insulin resistance is mainly initiated and sustained by inflammation in adipose tissue, caused by the infiltration of immune cells, such as macrophages, surrounding hypertrophied adipocytes [1, 2]. Consumption of a high-fat diet (HFD) changes the gut microbiota, which induces endotoxemia followed by chronic inflammation in various tissues, including adipose tissue [3]. Insulin resistance caused by obesity is often accompanied by a transient increase in insulin secretion and the number of pancreatic β-cells as a compensatory mechanism. However, this compensation cannot be sustained over time, leading to the attenuation of insulin secretion and β-cell mass reduction, which ultimately induces diabetes [4, 5].

Apoptosis is an important mechanism responsible for the decrease in β-cell mass. Chronic hyperglycemia or free fatty acids (FFAs) have been shown to induce increased endoplasmic reticulum stress, reactive oxygen species, and amyloid deposition, thereby promoting β-cell apoptosis [6–9]. Further, p53-mediated pathway is reportedly associated with β-cell apoptosis [10]. However, the mechanism of β-cell apoptosis is not fully understood. Elucidation of the mechanisms involved in islet β-cell mass reduction is essential for establishing fundamental treatment strategy against the onset and progression of diabetes.

Infiltration of inflammatory cells, such as macrophages, is observed in the islets of patients with type 2 diabetes mellitus [11], although the influence of these inflammatory cells on β-cell mass reduction remains unclear. We previously reported that activated macrophages stimulated with toll-like receptor 4 ligand promoted the secretion of interferon (IFN) β. Furthermore, IFNβ-stimulated β-cells significantly increased the expression of an IFN-inducible gene, *X-linked inhibitor of apoptosis–associated factor 1* (*Xaf1*) [12]. XAF1 promotes apoptosis by inhibiting X-linked inhibitor of apoptosis protein (XIAP), which inhibits caspase-3 activation and suppresses the apoptosis cascade [13].

In our previous work, we reported that endotoxin-stimulated macrophages produced IFNβ, which resulted in increased XAF1 protein production in βTC6 cells and accelerated β-cell apoptosis when exogenously added to the cell culture [12]. However, the in vivo effects of inflammation-induced XAF1 on β-cell function warranted further investigation. Here, we generated transgenic mice selectively expressing the *Xaf1* gene in β-cells, and examined the effects of β-cell XAF1 on β-cell function and the development of diabetes in vivo. The study aimed to determine the mechanism of pancreatic β-cell mass reduction as one of the fundamental pathological features of diabetes.

## Methods

### Animals

Transgenic (Tg) founder mice (F0) expressing the *Xaf1* gene selectively in pancreatic β-cells were generated using *pRIP-N-Myc-Xaf1* expression vector (Supplementary Fig. 1a). Diets fed the mice were the same as those used in the previous report [14], as described in the supplementary information. The animal study was reviewed and approved by Institutional Animal Care and Use Committee of the University of Kyushu (protocol #A20-101–0 and #1–7).

### Islet isolation and insulin secretion assay

Islet isolation and insulin secretion assay was performed as described previously [15]. Islets were isolated by perfusing the pancreatic duct with Collagenase P (Roche, Basel, Switzerland), followed by digestion for 12 min at 37 °C. For the insulin secretion assay, 0.5 mL of RPMI 1640 medium containing 3 mM glucose (pH 7.4) was added to ten purified islets, and then the suspension was incubated at 37 °C for 60 min under 5% CO^2^. After centrifugation, the supernatant was removed. To the residue (the precipitated islets), 0.2 mL of 3 mM glucose in RPMI 1640 medium was added, and the suspension was incubated at 37 °C for 60 min under 5% CO^2^. The suspension was centrifuged again and the supernatant was collected for analysis as the sample was stimulated with 3 mM glucose. To the residue, a mixture containing 0.2 mL of 20 mM glucose was added and incubated at 37 °C for a further 60 min under 5% CO^2^. The suspension was centrifuged and the supernatants were collected for analysis as the sample stimulated with 20 mM glucose. The insulin levels in the supernatant were measured using a Mouse Insulin ELISA Kit. The data were normalized by the protein concentration of the lysate.

### Immunofluorescence staining

Immunofluorescence staining was performed as described previously [14] using the antibodies listed in the supplementary information. Paraffin-embedded tissues of mice samples were cut into 5 µm sections and they were dewaxed in xylene and rehydrated in graded ethanol solutions. Antigen retrieval was performed with citrate buffer (6 pH) for 10 min at 95 °C. Sections rinsed in PBS for 5 min. Non-specific labeling was blocked by incubation with 1% BSA at room temperature for 30 min. Sections were then incubated with primary antibodies at 4 °C overnight. After incubation with the primary antibody, the sections were washed and incubated with secondary antibodies for 2 h in the dark at room temperature. The samples were visualized by Keyence BZ-9000 (Keyence, Osaka, Japan). Positive areas were quantified using Image J software (National Institute of Health, Bethesda, MA, USA). Twenty islets per experimental group (4 animals in each group and 5 random islets per animal) were evaluated in each analysis. The sizes of β-cell and α-cell area per pancreas section were calculated from 15 sections (random 3 to 4 sections from 4 animals in each group).

### TUNEL staining

Evaluation of apoptosis was performed with ApopTag® Plus In Situ Apoptosis Fluorescein Detection Kit (Merck, Darmstadt, Germany) to find DNA strand breaks using the terminal deoxynucleotidyl transferase-mediated dUTP nick end labeling (TUNEL) reagent. According to the manufacturer's instructions, TUNEL staining was performed in combination with insulin immunofluorescence staining. The samples were visualized by Keyence BZ-9000 (Keyence). The average number of TUNEL positive cells per islet of 5 random fields from each sample were quantitated using Image J software (National Institute of Health).

More detailed methods are described in the Supplementary Information.

## Results

### Palmitic acid (PA) promoted IFNβ secretion from macrophages

**Fig. 1:**
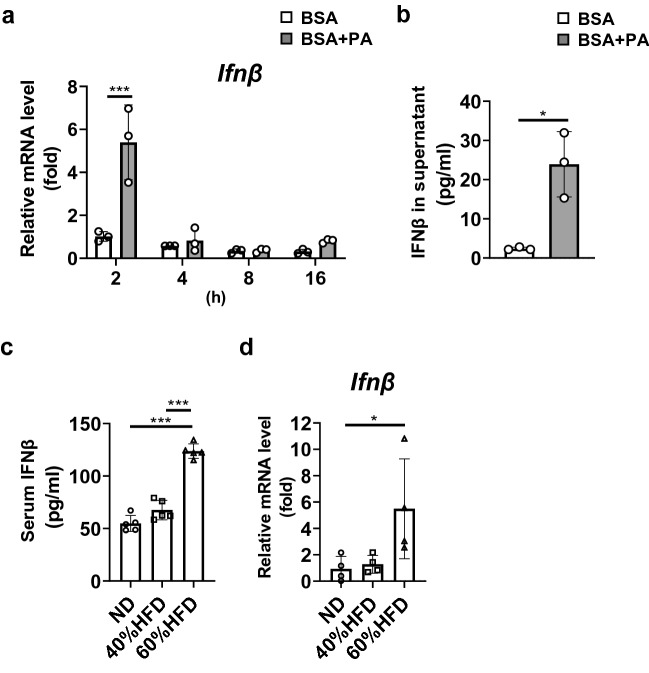
Palmitic acid promoted IFNβ secretion from macrophages. **a** *Ifnβ* mRNA expression levels following 2, 4, 8, and 16 h stimulation with palmitic acid (PA) or bovine serum albumin (BSA) vehicle in RAW264.7 macrophages. Expression fold changes relative to *18S* mRNA were determined by RT-PCR (*n* = 3). **b** IFNβ protein concentration in the culture supernatants was determined 24 h after stimulation. **c, d** C57BL/6N mice were fed a normal diet (ND), 40% high-fat diet (HFD), or 60%HFD for 10 weeks. **c** IFNβ protein concentration in mouse serum was determined by ELISA kits. **d** *Ifnβ* mRNA expression levels in the islet of mice. Representative data from three independent experiments are shown. Data are expressed as mean (SD). *p < 0.05, ***p < 0.001

We previously reported that *Ifnβ* mRNA expression and IFNβ secretion were increased in RAW264.7 macrophages stimulated with lipopolysaccharide [12]. Obese individuals have elevated blood concentration of saturated fatty acids, such as PA. Thus, we stimulated macrophages with PA. *Ifnβ* mRNA expression was significantly upregulated in PA-stimulated RAW264.7 macrophages compared with that in vehicle-stimulated controls (Fig. 1a). IFNβ secretion from RAW264.7 macrophages was also increased after 24-h stimulation with PA (Fig. 1b). We also investigated the effects of HFD feeding on systemic IFNβ levels in vivo. The serum IFNβ concentration was significantly higher in 60%HFD-fed mice than in mice ND and 40%HFD groups (Fig. 1c). *Ifnβ* mRNA expression level in the islet of 60%HFD-fed mice was significantly elevated compared with ND-fed mice (Fig. 1d).

### High-fat diet intake induced XAF1 expression in mouse islets

**Fig. 2:**
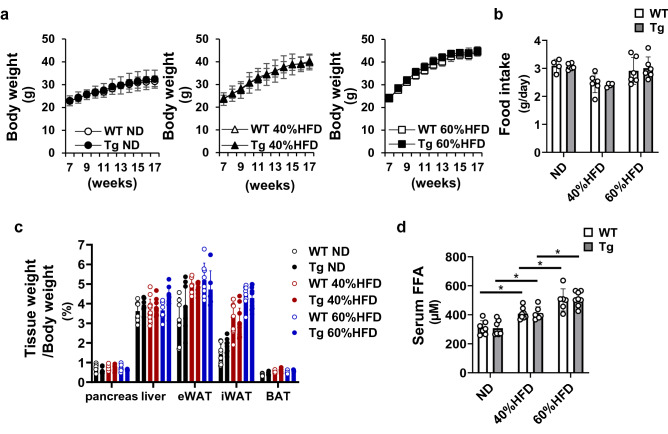
Characteristics of *Xaf1* Tg mice. Wild-type (WT) and *Xaf1* Tg (Tg) mice were fed with a normal diet (ND) up to 7 weeks of age after weaning. From 7 weeks of age, mice were fed an ND, a 40%, or a 60% high-fat diet (40%HFD, 60%HFD) for 10 weeks. **a** Changes in body weight. **b** Mean daily food intake. **c** Tissue weight. **d** Serum free fatty acid (FFA) concentration. (*n* = 5–8 per group) Data are expressed as mean ± SD. *p < 0.05. eWAT, epididymal white adipose tissue; iWAT, inguinal white adipose tissue; BAT, brown adipose tissue

**Fig. 3:**
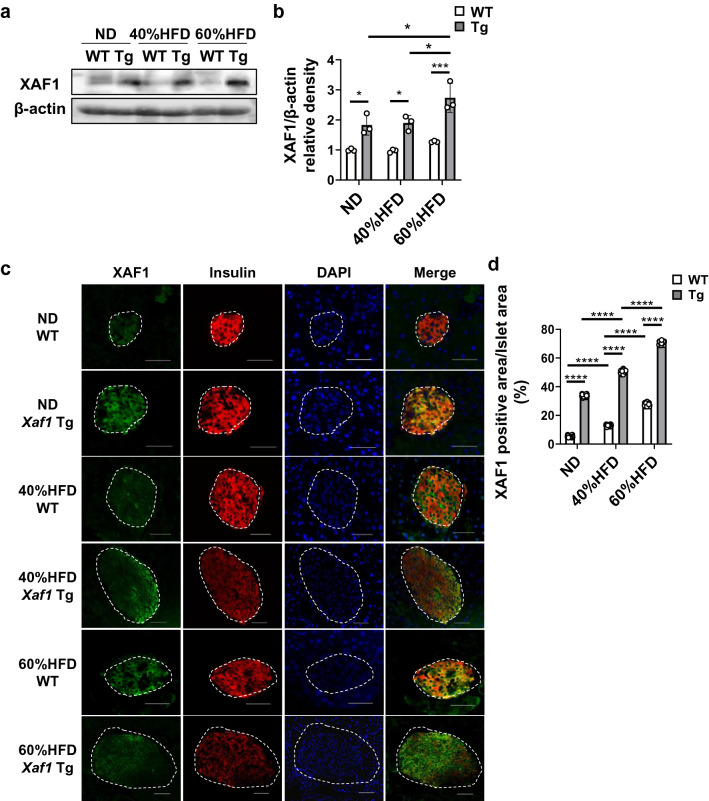
High-fat diet intakes induced XAF1 expression in mouse islets. Wild-type (WT) and *Xaf1* Tg (Tg) mice were fed with a normal diet (ND) up to 7 weeks of age after weaning. From 7 weeks of age, mice were fed an ND, a 40%, or a 60% high-fat diet (40%HFD, 60%HFD) for 10 weeks. **a** Western blot analysis of XAF1 expression in islets of mice. Representative blots are shown. **b** Quantitative data of each corresponding western blot images from 3 independent experiments were presented as bar graphs. **c** Representative images of immunofluorescence staining for XAF1 (green) and insulin (red) in pancreatic sections. Areas surrounded by white dotted lines indicate islets. The double stained area for XAF1 and insulin is shown in yellow. Scale bar = 50 µm, 40 × magnification. **d** Quantification of the average XAF1-positive area per islet area. Four animals in each group and 5 random islets per animal were quantified. Data are expressed as mean ± SD. *p < 0.05, ***p < 0.001, ****p < 0.0001 (color figure online)

We previously performed comprehensive gene expression analysis in αTC1 and βTC6 cells individually co-cultured with RAW264.7 macrophages [12]. *Xaf1* gene expression was elevated by LPS stimulation in βTC6 cells, whereas almost no change was observed in αTC1 cells. In addition, IFNβ stimulation to βTC6 cells resulted in increased XAF1 protein production, cleaved caspase-3 expression, and β-cell apoptosis [12]. Based on these results, we generated β-cell-selective XAF1 overexpressing (*Xaf1* Tg) mice to investigate the role of β-cell XAF1 in vivo. We confirmed that the XAF1 protein level was upregulated in islets of *Xaf1* Tg mice, whereas XAF1 expression levels were unchanged in the liver and epididymal adipose tissues (Supplementary Fig. 1b). It has been reported that the RIP promoter results in widespread expression in the hypothalamus [16]. To confirm the specificity of our overexpression system, XAF1 expression level in the hypothalamus was validated (Supplementary Fig. 1c). In our model, there seemed to be little effect on XAF1 expression in the hypothalamus of *Xaf1* Tg mice. Within the same diet group (normal diet [ND], 40% or 60% HFD), values for total body weight, food intake, and tissue weight did not differ significantly between *Xaf1* Tg mice and their WT littermates (Fig. 2a–c). At 17 weeks of age, WT mice gained 26.2% in the 40%HFD group and 41.5% in the 60%HFD group compared with the ND group, respectively. Also, at 17 weeks of age, *Xaf1* Tg mice gained 24.2% in the 40%HFD group and 38.4% in the 60%HFD group compared with the ND group, respectively. Serum FFA levels were increased in both WT and *Xaf1* Tg mice depending on the fat content of the diet, and no significant differences were observed between genotypes in each diet group (Fig. 2d). XAF1 expression was enhanced in the islets of *Xaf1* Tg mice compared with those of WT mice (Fig. 3a–3d). In *Xaf1* Tg mice, the XAF1-positive area increased proportionally with the amount of dietary fat (Fig. 3d). In WT mice, XAF1 expression tended to be promoted in the 60%HFD group (Fig. 3d). The double stained area for XAF1 and insulin is shown in yellow (Fig. 3c). Many XAF1- and insulin-positive cells were observed in the islets of *Xaf1* Tg mice in the ND group (Fig. 3c). However, *Xaf1* Tg mice in the 60%HFD group exhibited reduced insulin expression and, thus, increased numbers of XAF1-positive and insulin-negative cells in the islets (Fig. 3c). Although few XAF1-positive cells were observed in the islets of ND-fed WT mice, the XAF1-positive area was increased in HFD-fed WT mice (Fig. 3b, d). *Xiap* gene expression levels did not differ significantly between the groups (data not shown). This observation is consistent with previously reported results in other studies, in which XAF1 expression did not influence XIAP expression [17].

### High-fat diet-fed Xaf1 Tg mice exhibited attenuated insulin expression and impaired glucose tolerance

**Fig. 4:**
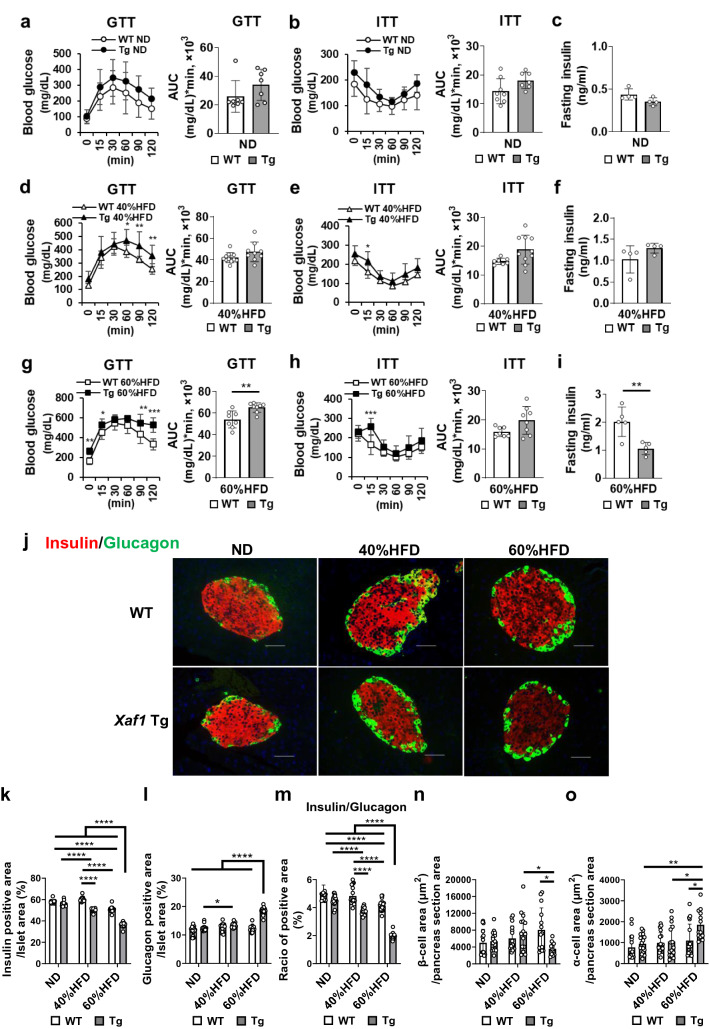
High-fat diet-fed *Xaf1* Tg mice displayed attenuated insulin expression and impaired glucose tolerance compared with WT mice fed the same diet. Wild-type (WT) and *Xaf1* Tg (Tg) mice were fed with a normal diet (ND) up to 7 weeks of age after weaning. From 7 weeks of age, mice were fed an ND, a 40%, or a 60% high-fat diet (40%HFD, 60%HFD) for 10 weeks. **a, d, g** Glucose tolerance test (GTT) was performed in each group (**a**: *n* = 7 [WT, *Xaf1* Tg], **d**: *n* = 12 [WT], 9 [*Xaf1* Tg], **g**: *n* = 8 [WT, *Xaf1* Tg]). **b, e, h** Insulin tolerance test (ITT) was performed in each group (**b**: *n* = 8 [WT], 7 [*Xaf1* Tg], **e**: *n* = 7 [WT], 10 [*Xaf1* Tg], **h**: *n* = 7 [WT], 8 [*Xaf1* Tg]). AUC, area under the curve. **c, f, i** Fasting insulin levels in each test group (**c, f**: *n* = 4 [WT, *Xaf1* Tg], **i**: *n* = 5 [WT, *Xaf1* Tg]). **j** Representative images of immunofluorescence staining for insulin (red) and glucagon (green) of pancreatic sections. Scale bar = 50 µm, 40 × magnification. **k–m** Quantification of the average **(k)** insulin- and **(l)** glucagon-positive areas per islet area. Quantification of average **(m)** ratio of insulin- to glucagon-positive areas per islet. **n**, **o** Quantification of the average **(n)** β-cell and **(o)** α-cell area per pancreas section. Data are expressed as mean ± SD. Four animals in each group and 5 random islets per animal were quantified. *p < 0.05, **p < 0.01, ***p < 0.001, ****p < 0.0001 (color figure online)

Fasting insulin levels and blood glucose levels during the glucose tolerance test (GTT) and insulin tolerance test (ITT) did not differ significantly between ND-fed WT and *Xaf1* Tg mice (Fig. 4a–c). However, in the 40% and 60% HFD groups, fasting glucose levels were significantly higher in *Xaf1* Tg mice than in WT mice (Fig. 4d, g). In addition, the decline in blood glucose levels after reaching the peak was significantly delayed in *Xaf1* Tg mice compared with WT mice in the 40% and 60% HFD groups (Fig. 4d, g). In particular, *Xaf1* Tg mice in the 60%HFD group demonstrated remarkably impaired glucose tolerance and significantly increased GTT-area under the curve (AUC) values compared with WT mice (Fig. 4g). In the ITT, *Xaf1* Tg mice in the 40% and 60% HFD groups displayed suppressed reduction of blood glucose levels 15 min after insulin administration compared to WT mice fed the same diets (Fig. 4e, h). In the 60%HFD group, fasting insulin levels and values of homeostasis model assessment of β-cell function, an index of insulin secretory capacity, were significantly lower in *Xaf1* Tg mice than in WT mice (Fig. 4i, Supplementary Fig. 2). The islets of *Xaf1* Tg mice in the 60%HFD group exhibited a markedly decreased insulin-positive area, increased glucagon-positive area, and decreased ratio of insulin- to glucagon-positive areas (Fig. 4j–m), compared with those in the other diet groups. The islets of 40%HFD-fed *Xaf1* Tg mice displayed a significantly reduced insulin-positive area and ratio of insulin- to glucagon-positive areas compared with those in 40%HFD-fed WT and ND-fed *Xaf1* Tg mice (Fig. 4k, m). The insulin and glucagon positive area per islet area was quantified as previously reported [18, 19]. The insulin-positive area and ratio of insulin- to glucagon-positive areas were significantly decreased in the islets of WT mice in the 60%HFD group compared with those in the 40%HFD and ND groups (Fig. 4k, m). The β-cell area per pancreas section of 60%HFD-fed *Xaf1* Tg mice was significantly decreased compared with that of 60%HFD-fed WT mice. On the other hand, α-cell area per pancreas section of the group of 60%HFD-fed *Xaf1* Tg mice was significantly increased compared with those of 60%HFD-fed WT mice and other dietary groups of *Xaf1* Tg mice (Fig. 4n, o). Hematoxylin and eosin staining of the pancreatic tissues revealed decreased cell density in the islet area of *Xaf1* Tg mice in the 60%HFD group compared with those in the other five groups (Supplementary Fig. 3). No significant differences in islet size distribution were observed between WT and *Xaf1* Tg mice fed the same diet (Supplementary Fig. 4).

### ***High-fat diet intake increased CD68***^+^***and Ly6C***^+^***cells in the islets of both WT and Xaf1 Tg mice***

Macrophage abundance is reportedly increased in the islets of mice fed HFD [20]. To examine the degree of macrophage infiltration in the islets, flow cytometric analysis and immunofluorescence staining was performed on islets isolated from the pancreatic tissues of mice in each group. Few CD68^+^ macrophages and Ly6C^+^ inflammatory macrophages were observed in the islets of WT and *Xaf1* Tg mice in the ND group (Supplementary Fig. 5a–c). The number of CD68^+^ cells increased in WT and *Xaf1* Tg mice in the 40% and 60% HFD groups (Supplementary Fig. 5a–c). In the 60%HFD group, the number of CD68^+^Ly6C^+^ cells in the islets increased to almost the same extent in both WT and *Xaf1* Tg mice (Supplementary Fig. 5c).

### High-fat diet intake enhanced β-cell apoptosis in Xaf1 Tg mice compared with WT mice

**Fig. 5:**
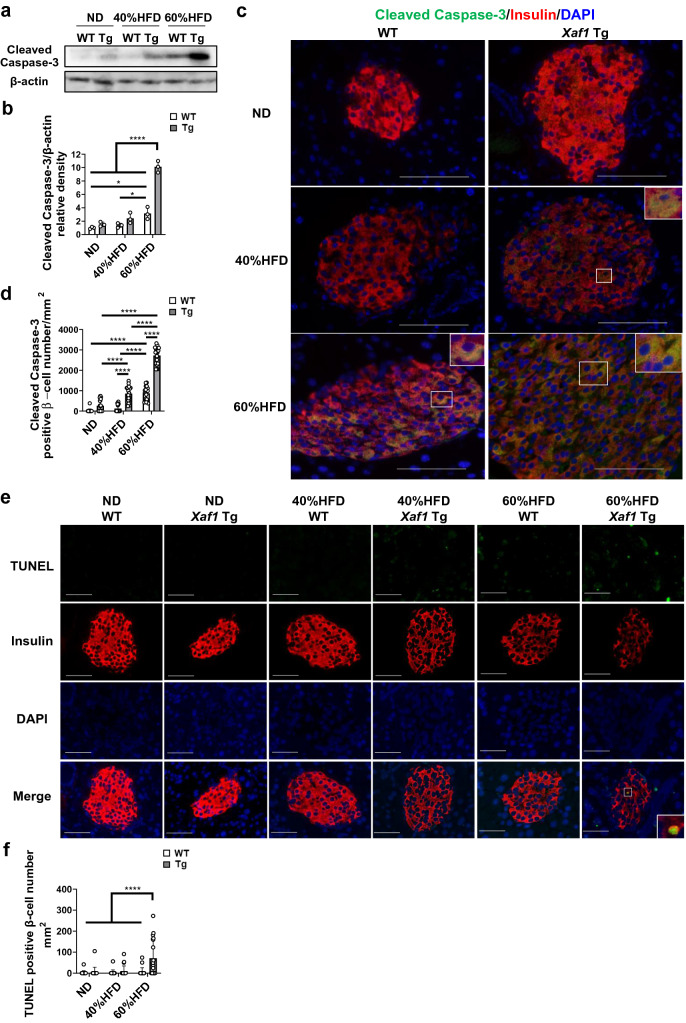
High-fat diet intake enhanced β-cell apoptosis in *Xaf1* Tg mice compared with WT mice. Wild-type (WT) and *Xaf1* Tg (Tg) mice were fed with a normal diet (ND) up to 7 weeks of age after weaning. From 7 weeks of age, mice were fed an ND, a 40%, or a 60% high-fat diet (40%HFD, 60%HFD) for 10 weeks. **a** Western blot analysis of cleaved caspase-3 expression in islets of mice. Representative blots are shown. **b** Quantitative data of each corresponding western blot images from 3 independent experiments were presented as bar graphs. **c** Double immunofluorescence staining for cleaved caspase-3 (green) and insulin (red) in pancreatic sections. The double stained area for cleaved caspase-3 and insulin is shown in yellow. The magnified inset images indicate double stained β-cells. **d** The number of cleaved caspase-3 positive β-cells per islet area was quantified. Four animals in each group and 5 random islets per animal were quantified. **e** Representative images in each group via combined terminal deoxynucleotidyl transferase-mediated dUTP nick end labeling (TUNEL) staining and insulin immunofluorescence staining in pancreatic sections. The magnified inset image indicates a β-cell with TUNEL-positive nuclei and insulin-positive cytosol. **f** The number of TUNEL positive β-cells per islet area was quantified. Four animals in each group and 5 random islets per animal were quantified. Scale bar = 50 µm, 40 × magnification. Data are expressed as mean (SD). *p < 0.05, ****p < 0.0001 (color figure online)

We next examined the effects of XAF1 expression on apoptosis. Caspase-3 is a principal enzyme in the process of apoptosis. The islets of *Xaf1* Tg mice in the 60%HFD group exhibited a marked increase of cleaved caspase-3 protein expression compared with the other diet groups (Fig. 5a, b). In the islets of 60%HFD-fed WT mice, expression level of cleaved caspase-3 was significantly increased compared with that in the islets of WT mice in the 40%HFD or ND groups (Fig. 5a, b). After the expression of cleaved caspase-3, cells irreversibly undergo apoptosis. Detection of cleaved caspase-3 enables to recognize apoptosis earlier than the stage of DNA strand breaks. Thus, immunohistochemical detection of activated caspase-3 is a useful method for identification of early apoptosis in tissue sections [21]. To detect β-cell apoptosis, double immunofluorescence staining for cleaved caspase-3 and insulin was performed and the double-positive areas were quantified (Fig. 5c, d, Supplementary Fig. 6a, b). Since both cleaved caspase-3 and insulin are expressed in the cytosol, yellowish staining indicates co-localization. As shown in the magnified inset images, β-cells with insulin and cleaved caspase-3-positive cytosol were significantly increased in the islets of *Xaf1* Tg mice in the HFD groups compared with those of WT mice, which was proportional to the amount of dietary fat (Fig. 5c, d). In the islets of WT mice in the 60%HFD group, the cleaved caspase-3-positive β-cells were significantly increased compared to that in the islets of WT mice in the 40%HFD or ND groups (Fig. 5d). Thus, significantly increased β-cell apoptosis was observed in the HFD-fed group with enhanced XAF1 expression levels. To further confirm β-cell apoptosis, combined TUNEL staining and insulin immunofluorescence were conducted (Fig. 5e, f, Supplementary Fig. 7). As shown in the magnified inset image, β-cells with TUNEL-positive nuclei and insulin-positive cytosol were observed in the islets of *Xaf1* Tg mice in the 60%HFD group (Fig. 5e). The number of TUNEL-positive β-cells was fewer than cleaved caspase-3 positive cells, yet significantly increased in the pancreatic islets of *Xaf1* Tg mice in the 60%HFD group compared with the other groups (Fig. 5f). TUNEL detects DNA strand breaks in the late stage of apoptosis. However, cells with necrotic morphology are often stained vaguely [22, 23], or DNA fragmentation is even absent or incomplete in induced apoptosis [24]. Therefore, our observation is consistent with the previous observation reporting the difficulty of accurately detecting TUNEL and insulin double positive cells [25].

### Xaf1 Tg mice fed 60%HFD showed markedly impaired insulin secretion

**Fig. 6:**
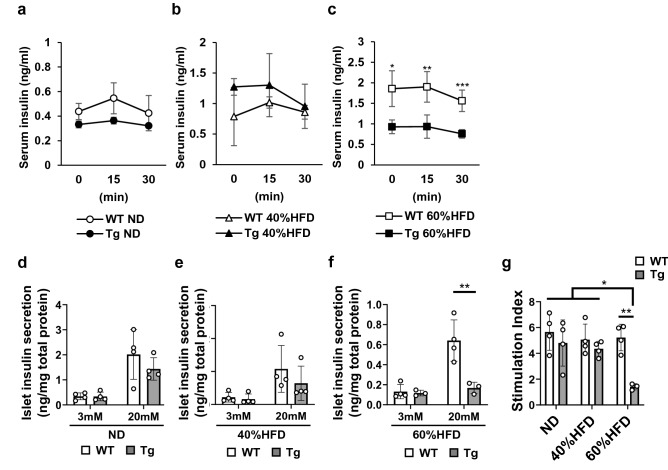
*Xaf1* Tg mice fed 60%HFD showed markedly impaired insulin secretion. Wild-type (WT) and *Xaf1* Tg (Tg) mice were fed with a normal diet (ND) up to 7 weeks of age after weaning. From 7 weeks of age, mice were fed an ND, a 40%, or a 60% high-fat diet (40%HFD, 60%HFD) for 10 weeks. **a, b, c** Insulin response to intraperitoneal glucose administration (2 g/kg body weight). **d, e, f** Basal and glucose-stimulated insulin secretion in islets isolated from WT and *Xaf1* Tg mice. **g** Simulation index (SI) values. SI was calculated by dividing insulin concentrations at high (20 mM) glucose by that at low (3 mM) glucose. Representative data from three independent experiments are shown (*n* = 3–4 per group). Data are expressed as mean (SD). *p < 0.05, **p < 0.01, ***p < 0.001

The GTT revealed markedly impaired glucose tolerance in *Xaf1* Tg mice compared with WT mice in the 60%HFD group (Fig. 4g). Subsequently, we investigated serum insulin levels after glucose administration to evaluate the effect of XAF1 overexpression on insulin secretion capacity. In the ND and 40%HFD groups, serum insulin levels did not differ significantly after glucose administration between WT and *Xaf1* Tg mice (Fig. 6a, b). In contrast, serum insulin levels after glucose administration were markedly lower in *Xaf1* Tg mice than in WT mice in the 60%HFD group (Fig. 6c). To confirm the in vivo results, an insulin secretion assay was performed using organ-cultured pancreatic islets isolated from WT and *Xaf1* Tg mice (Fig. 6d, e, f) and calculated the stimulation index (Fig. 6g). Consistent with in vivo results, in the 60%HFD group, insulin secretion in *Xaf1* Tg mouse islets was severely impaired compared to that in WT mice upon stimulation with high doses of glucose (Fig. 6f, g).

## Discussion

In this study, we demonstrated that macrophage expression of IFNβ upon PA stimulation and intake of HFD promoted macrophage infiltration in pancreatic islets, thereby inducing increased XAF1 expression in mice. We previously revealed that IFNβ-induced XAF1 expression was elevated in β-cells in a receptor-dependent manner [12]. Collectively, the results confirmed that XAF1 expression in pancreatic β-cells was enhanced by consumption of HFD. Furthermore, HFD-fed *Xaf1* Tg mice demonstrated increased β-cell apoptosis, attenuated insulin expression, and impaired glucose tolerance compared with WT mice fed the same diet. These effects were more pronounced in 60%HFD group than in the 40%HFD-fed groups. These results clarified that HFD-induced enhancement of IFNβ production in macrophages augments XAF1 expression in pancreatic β-cells, promotes β-cell apoptosis, and ultimately reduces insulin secretion. Serum IFNβ levels were significantly increased in 60%HFD fed mice compared with those of ND and 40%HFD fed mice (Fig. 1c). Previously, it has been reported that correlation between systemic IFNβ and triglyceride levels, and type I IFN/IFNAR axis may be positively correlated with individuals with obesity-associated hepatocellular disease [26]. To the best of our knowledge, this is the first study to demonstrate an association between HFD-induced pancreatic β-cell XAF1 expression and exacerbation of diabetes.

The effects of excess levels of circulating lipids, called lipotoxicity, on β-cell survival appear to be regulated by several distinct mechanisms. Several mediating factors of apoptosis have been suggested. The roles of endoplasmic reticulum stress in lipotoxicity have been demonstrated in multiple cell types, including β-cells [27, 28]. Jeffrey et al. reported that carboxypeptidase E degradation contributed to the elevation of palmitate-induced β-cell endoplasmic reticulum stress and apoptosis [27]. As shown in this study, β-cell apoptosis via the IFNβ-XAF1 pathway was initiated from increased IFNβ production by macrophages. IFNβ secretion was upregulated in RAW264.7 macrophages upon PA stimulation (Fig. 1b). It cannot be ruled out that RAW264.7 macrophages may not respond like primary macrophages. *Ifnβ* mRNA levels were significantly elevated in the islet of 60%HFD fed mice compared with ND fed mice (Fig. 1d). Considering the increase of the number of macrophages in islets of mice fed a high-fat diet, the source of the elevated *Infβ* expression in islets may be macrophages.

Obesity-induced inflammation, characterized by an increased number of proinflammatory macrophages in adipose tissue, has been suggested to contribute to systemic insulin resistance [29]. Obesity-induced infiltration of macrophages has also been observed in pancreatic islets. β-cells reportedly secrete chemokines such as Monocyte chemotactic protein 1 and chemokine (C-X-C motif) ligand 1 in response to toll-like receptor 4 ligand, PA, or lipopolysaccharide, all of which recruit proinflammatory macrophages into islets [12, 20].

The present study demonstrated that low-grade inflammation that causes macrophage activation under obesity conditions induces β-cell dysfunction and exacerbates diabetes due to enhanced β-cell apoptosis mediated by XAF1 expression, in addition to decreased insulin sensitivity through adipose tissue inflammation. This may be a newly clarified mechanism that causes an obesity-induced decrease in pancreatic β-cell mass. *Xaf1* Tg mice fed a 60%HFD displayed remarkably impaired glucose tolerance and progression of β-cell apoptosis compared to *Xaf1* Tg mice fed 40%HFD and ND. This result supports the hypothesis that elevated glucose and lipid levels synergistically induces a more detrimental glucolipotoxic state [30].

Several reports have suggested the critical involvement of IFNβ and XAF1 in apoptosis. Enhancement of XAF1 expression by IFNβ and subsequent apoptosis via the IFNβ-XAF1 pathway has been reported in a colon cancer cell line and in germ cells [31, 32]. Other studies have demonstrated that XAF1 mediates apoptosis via the mitochondrial and p53 pathways, independently of XIAP interaction [33–35]. XAF1 plays a role as an apoptosis-promoting tumor suppressor. In human malignancies, XAF1 expression is reportedly inactivated by aberrant promoter methylation and subsequent suppression of critical gene expression [36, 37]. Our previous study confirmed that *Xaf1*-knockdown attenuated IFNβ-induced apoptosis in a mouse pancreatic β-cell line in vitro [12]. Therefore, targeting excess XAF1 expression in pancreatic β-cells may provide a therapeutic objective to prevent β-cell depletion in diabetes. However, direct suppression of XAF1 may influence the tumorigenesis signal of β-cells. Further verification of mechanisms such as IFNβ signaling leading to XAF1 expression is required for clinical applications.

In conclusion, the present study clearly demonstrates that XAF1 expression in pancreatic β-cells via HFD-induced secretion of IFNβ from macrophages promoted β-cell apoptosis, thereby leading to a reduction in insulin secretion and exacerbation of diabetes. The study findings provide valuable insight into the mechanism of pancreatic β-cell mass reduction, a fundamental pathological feature of diabetes, to help establish therapeutic strategies against diabetes.

## Supplementary Information

Below is the link to the electronic supplementary material.

Supplementary file1 (DOCX 5054 kb)
