# Supplementary material for: XAF1 overexpression exacerbates diabetes by promoting pancreatic β-cell apoptosis
Source: Acta Diabetol. 2022 Jul 13;59(10):1275–86. doi: 10.1007/s00592-022-01930-y (PMC9402739; doi:10.1007/s00592-022-01930-y)
Supplement: Supplementary file 1 — Supplementary file1 (DOCX 5054 kb) [file 592_2022_1930_MOESM1_ESM.docx]

**Title:** XAF1 overexpression exacerbates diabetes by promoting pancreatic β-cell apoptosis

**Journal name:** Acta Diabetologica

**Authors:** Yuki Nishimura, Misaki Iwashita, Masato Hayashi, Takanori Shinjo, Yukari Watanabe, Tatsuro Zeze, Akiko Yamashita, Takao Fukuda, Terukazu Sanui, Tomomi Sano, Tomoichiro Asano, and Fusanori Nishimura

**Corresponding author:** Misaki Iwashita

**Affiliation:** Department of Periodontology, Division of Oral Rehabilitation, Faculty of Dental Science, Kyushu University, Fukuoka, Japan

**E-mail:** iwashita.misaki.896@m.kyushu-u.ac.jp

**Materials and Methods**

**Cell culture**

Murine macrophage cell line RAW264.7 (American Type Culture Collection, Manassas, VA, USA) was maintained in Dulbecco’s modified Eagles medium (DMEM) (Gibco, Massachusetts, USA) containing 10% fetal bovine serum (Biowest, Nuaillé, France) in an atmosphere of 5% CO^2^ at 37°C.

**Palmitic acid (PA) treatment**

RAW264.7 cells (1×10^6^ cells/well) were treated with 100 µM PA or bovine serum albumin (BSA) vehicle for the indicated times as previously [1]. PA was solubilized in 0.1 N sodium hydroxide and combined with 10% fatty acid-free BSA. PA/BSA mixture was further diluted in DMEM to obtain the required final concentrations (a molar PA/BSA ratio of 3.3 as seen in extremes of fasting or in diabetic states).

**Animals and Diets**

C57BL/6N mice were purchased from Japan SLC (Shizuoka, Japan). Transgenic (Tg) founders (F0) were crossed with wild-type (WT) C57BL/6N mice to generate F1 Tg mice and subjected to western blotting to check XAF1 protein expression in pancreas. The F2 Tg offsprings were generated by crossing the F1 Tg mice with WT mice. Their male descendants were used in the experiments. *Xaf1* Tg mice and their WT littermates were fed a normal diet (13.6% fat content) (CRF-1, Oriental Yeast Co. Ltd., Tokyo, Japan) from the age of 4 weeks. In the experiments using high-fat diet (HFD), mice were fed a 40%HFD (a diet of 15% lard added to CRF-1) or a 60%HFD (HFD-60, Oriental Yeast Co. Ltd.) for 10 weeks from 7 weeks of age. Body weights were monitored and food intake was recorded by subtracting the amounts of food left in the cage from the daily amounts given. At 17 weeks of age, mouse tissue samples were collected following intraperitoneal injection of a mixed anesthetic (0.3 mg/kg medetomidine, 4.0 mg/kg midazolam, and 5.0 mg/kg). All mice were housed under climate-controlled conditions with a 12-hour light/dark cycle and with food and water ad libitum.

**Western blot analysis**

Tissue sections were solubilized with CytoBuster™ Protein Extraction Reagent (Millipore, Billerica, MA, USA). After adjusting the protein concentrations, the supernatants were mixed and boiled with sample buffer. Equal amounts of protein were resolved by SDS-polyacrylamide gel. Then, the proteins were transferred to polyvinylidene difluoride membranes (Millipore) using the semi-dry system (Trans-Blot🄬SD Semi-Dry Transfer Cell [Bio-Rad Laboratories, Hercules, CA, USA]). The membranes were incubated overnight at 4°C with specific antibodies: rabbit anti-XAF1 (polyclonal, 1:1000, ab17024, Abcam, Cambridge, UK), rabbit anti-cleaved caspase-3 ([Asp175] [5A1E], 1:1000, #9664, Cell Signaling Technology, Danvers, MA, USA), and mouse anti-β-actin (8H10D10, 1:5000, #3700, Cell Signaling Technology). After incubation with primary antibodies, membranes were incubated with HRP-tagged anti-mouse or anti-rabbit secondary antibodies (HRP-conjugated anti-rabbit and anti-mouse, Cell Signaling Technology) for 1 hour at room temperature. Immunoreactive proteins were visualized using enhanced chemiluminescence (Chemi-Lumi One Super, Nacalai Tesque, Kyoto, Japan) and signals were analyzed using Image Quant LAS4000 (GE Healthcare, Chalfont, UK).

**Glucose tolerance test (GTT) and insulin tolerance test (ITT)**

For GTT, mice were injected glucose (2 g/kg body weight) intraperitoneally after 16 hours of fasting at 15 weeks of age. For ITT, mice were injected insulin (0.75 U/kg body weight) intraperitoneally after 6 hours of fasting at 16 weeks of age. Blood was collected 15, 30, 60, 90, and 120 min after the injection. Blood glucose concentrations were determined with a glucometer (Medisafe, Terumo, Tokyo, Japan).

**Quantitative real-time PCR**

Total RNA was extracted from the cells and tissues homogenized in ISOGEN II (Nippon Gene, Tokyo, Japan) and reverse transcribed using Prime Script RT Master Mix (Takara Bio, Otsu, Japan). The protocol for the reverse transcription cycle was 42°C for 30 min and 95°C for 2 min. RT-PCR was performed using KAPA SYBR®FAST qPCR Kit (Nippon Genetics, Tokyo, Japan) and Step One Plus Real Time PCR System (Applied Biosystems, Foster City, CA, USA). PCR was carried out in two steps, the first at 95°C for 5 seconds and the second at 60°C for 30 seconds, which were then repeated 40 times. Relative mRNA genes were normalized to the *18s* mRNA level and relative expression levels were calculated using the ΔΔCt method. Primers are listed in Supplementary Table 1.

**Enzyme-linked immunosorbent assay (ELISA)**

Serum insulin levels and serum free fatty acid (FFA) levels were measured with Mouse Insulin ELISA Kit (Morinaga Institute of Biological Science) and Free Fatty Acid Assay Kit (Cell Biolabs, San Diego, CA, USA) according to the manufacturer’s instructions, respectively. Interferon β (IFNβ) protein concentration in the culture media and mouse serum was determined by ELISA kits (R&D Systems, Minneapolis, MN, USA) according to the manufacturer’s instructions. Absorbances were determined using a microplate reader (Multiskan™ FC, Thermo Fisher Scientific, Waltham, MA, USA).

**Histological analysis**

For histological experiments, the tail regions of the pancreatic tissue were analyzed. Pancreatic tissue samples were ﬁxed in 4% paraformaldehyde solution. The paraffin blocks were cut into 5 μm thick sections. Hematoxylin and eosin staining was performed by standard procedures. The samples were visualized by Keyence BZ-9000 (Keyence, Osaka, Japan). All images were analyzed by Keyence BZ-X Analyzer software (Keyence). Four animals in each group and 5 random islets per animal were quantified in histological analysis.

**Antibodies used for immunofluorescence**

Primary antibodies: rabbit anti-XAF1 (polyclonal, 1:500, ab17024, Abcam), mouse anti-insulin (2D11-H5, 1:500, sc-8033, Santa cruz biotechnology, Dallas, TX, USA), rabbit anti-glucagon (polyclonal, 1:500, 15954-1-AP, Proteintech Japan, Tokyo, Japan), rabbit anti-cleaved caspase-3 ( [Asp175] [5A1E], 1:500, #9664, Cell Signaling Technology), and rabbit anti-CD68 (E3O7V, 1:500, #97778, Cell Signaling Technology). As isotype control, Rabbit (DA1E) mAb IgG XP^®^ Isotype Control (1:500, #3900, Cell Signaling Technology) was used instead of anti-cleaved caspase antibody (Supplementary Fig. 6a). Secondary antibodies: Alexa Fluor 488 goat anti-rabbit IgG (ab150077, Abcam) and Alexa Fluor 647 goat anti-mouse IgG (ab150115, Abcam). The nucleus was stained using SlowFade Diamond Antifade Mountant with DAPI (Life Technologies, Waltham, MA, USA).

**Flow cytometry analysis**

Isolated islets were dispersed into individual cells by incubation in Ca^2+^ and Mg^2+^ free Hank’s solution containing 1.0 mg/ml trypsin for 3 min at 37°C. Dissociated islet cells were washed with stain buffer (BD Pharmingen, San Diego, CA, USA). Prior to the surface staining, Fc-block was performed using mouse TruStain FcX™ (BioLegend, San Diego, CA, USA) for 5 min at room temperature. Subsequently, cells were stained with PE anti-mouse CD45 (clone 30-F11) (cat#103105, Biolegend), PE/Cyanine7 anti-mouse Ly6C (clone HK1.4) (cat#128017, Biolegend), and PerCP/Cyanine5.5 anti-mouse CD68 (clone FA-11) (cat#137009, Biolegend). Isotype controls were used to confirm antibody specificity. Cells were incubated in the dark for 30 min at 4°C and analyzed using a BD FACSVerse flow cytometer (BD Biosciences, San Diego, CA, USA). The data were analyzed using FlowJo (BD Biosciences) software.

**Statistical Analysis**

Data are presented as mean ± SD. Data analyses were performed using GraphPad Prism 8 (GraphPad Software, Inc., La Jolla, CA). and JMP Pro 16 (SAS Institute Japan, Tokyo, Japan). Two-way ANOVA with Holm-Sidak correction was used to compare three or more groups or to analyze the statistical significance of the GTT and ITT. Other statistical comparisons of data from two groups were performed using Student’s t-test. Values of p < 0.05 were considered statistically significant.

**Supplementary Table 1 Primer sequences used for real-time PCR**

| Gene | Forward | Reverse |
| --- | --- | --- |
| *Ifnβ* | TTGAAGGTCCTGGCACAG | GAGGTTCAAGGTCTGCTGA |
| *18S* | GCTTAATTTGACTCAACACGGGA | AGCTATCAATCTGTCAATCCTGTC |

**Supplementary Fig. 1**

**a**


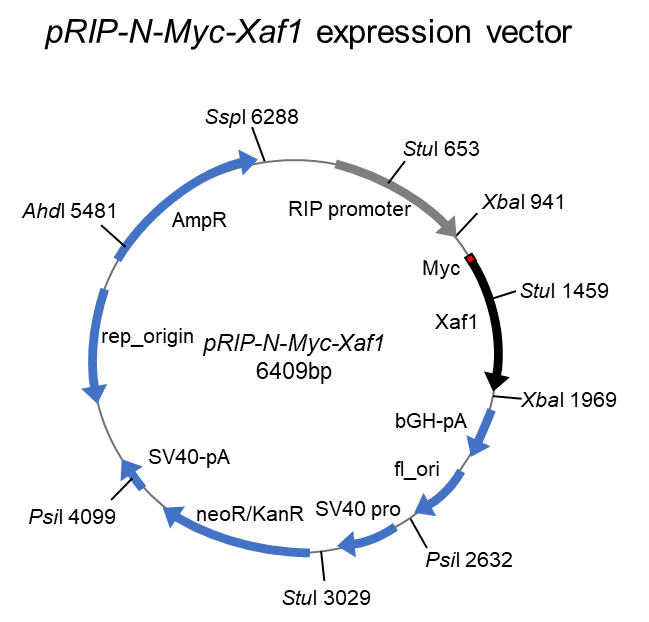


**b**
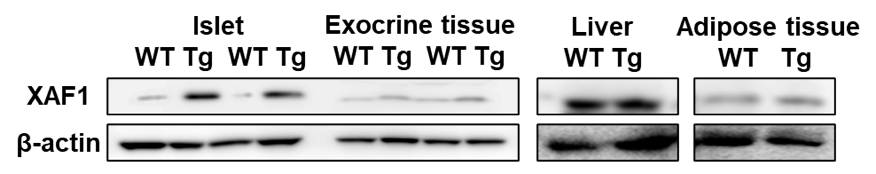


**c**
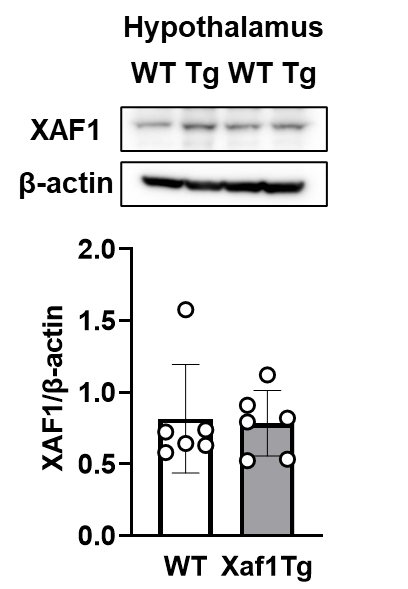


**a** *pRIP-N-Myc-Xaf1* expression vector constructed by replacing the promoter region of *pcDNA3.1-N-Myc-Xaf1* (GenScript Japan, Tokyo, Japan) with rat insulin II promoter (RIP) of RIP-Timer (Addgene, Watertown, MA, USA). The transgene was cut from the vector with SspI, PsiI, and AhdI restriction enzymes and microinjected into 228 fertilized eggs of C57BL/6N mice for founder mice production. For PCR screening, DNA was extracted from founder mice tail samples with KAPA Express Extract (Kapa Biosystems, Woburn, MA, USA). Primers for genotyping were TG-F2: 5′-AAAGTGCCACCTGACGTCGACG-3′ and TG-R2: 5′- AGCTGGTTCTTTCCGCCTCAGAAG-3′. All procedures of generation of founder mice were approved by Trans Genic Inc (Fukuoka, Japan). **b** Western blot analysis of XAF1 expression in islet, exocrine tissue, liver, and epididymal adipose tissue. **c** Western blot analysis of XAF1 expression in hypothalamus. Representative blots are shown. Quantitative data of each corresponding western blots images from 3 independent experiments were presented as bar graphs. WT, wild type; Tg, *Xaf1* Tg.

**Supplementary Fig. 2**

**
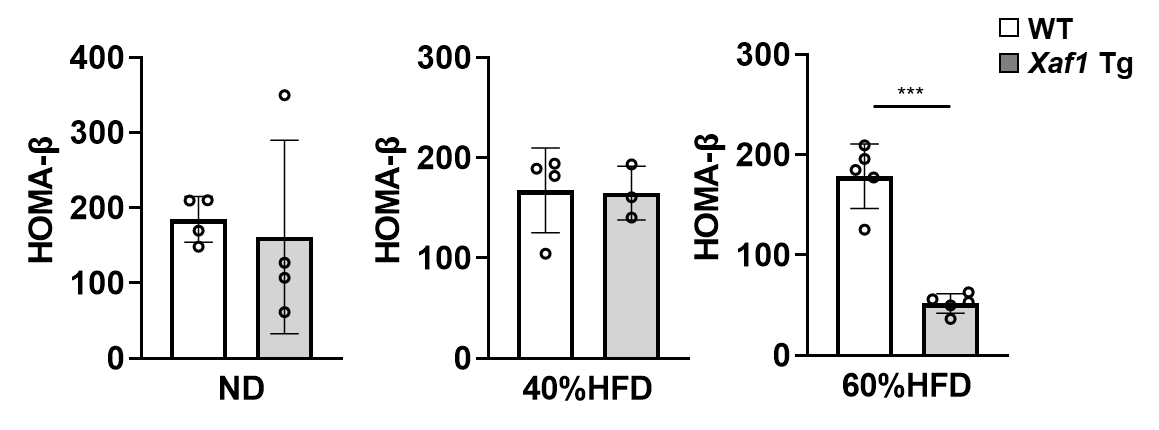
**

HOMA-β values were calculated using the equation (fasting insulin [μU/mL] × 360/fasting glucose [mg/dL]-63).

Wild-type (WT) and *Xaf1* Tg (Tg) mice were fed with a normal diet (ND) up to 7 weeks of age after weaning. From 7 weeks of age, mice were fed an ND, a 40%, or a 60% high-fat diet (40%HFD, 60%HFD) for 10 weeks. Data are expressed as mean ± SD (n = 3–5 per group). ***p < 0.001.

**
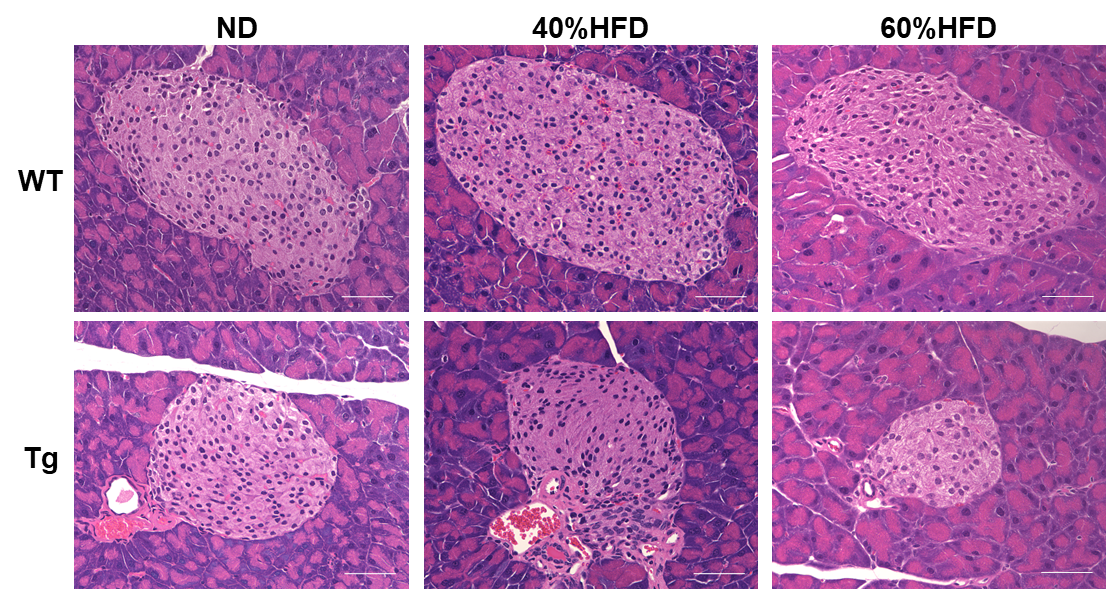
S****upplementary Fig. 3**

Representative images of hematoxylin and eosin stained pancreatic tissue sections.

Scale bar = 50 µm, 40 × magnification. Wild-type (WT) and *Xaf1* Tg (Tg) mice were fed with a normal diet (ND) up to 7 weeks of age after weaning. From 7 weeks of age, mice were fed an ND, a 40%, or a 60% high-fat diet (40%HFD, 60%HFD) for 10 weeks.

**Supplementary Fig. 4**


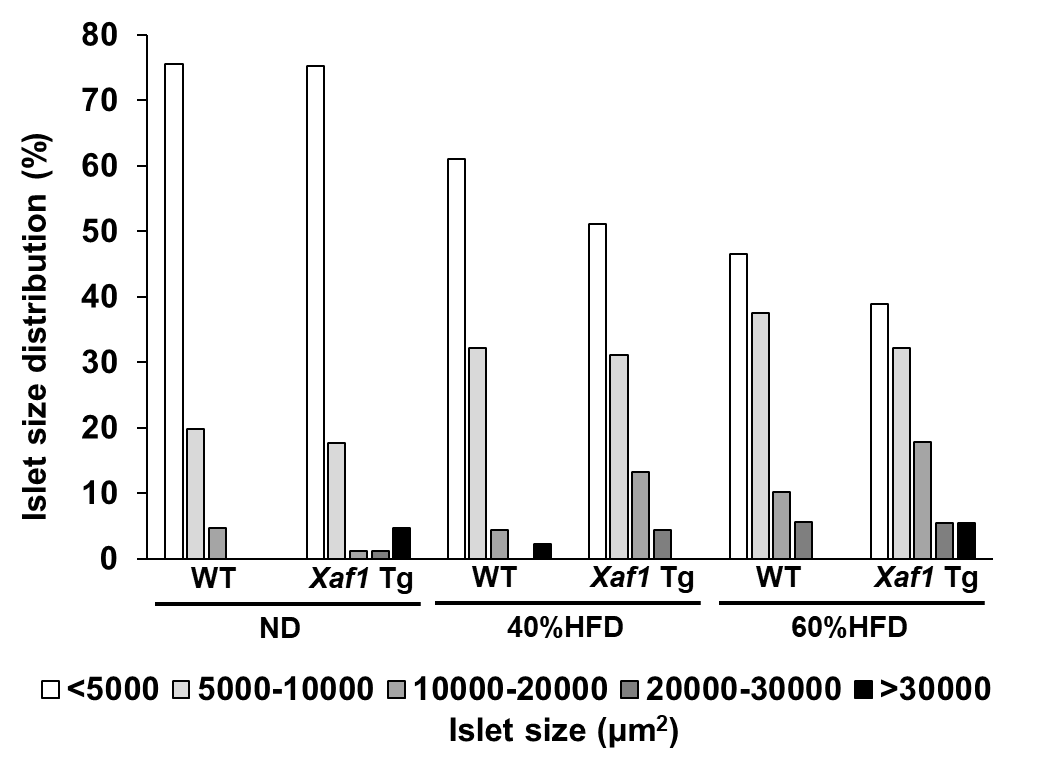


Distribution of islet size. The size of 85 to 90 random islets from 8 animals in each group was quantified.

Wild-type (WT) and *Xaf1* Tg (Tg) mice were fed with a normal diet (ND) up to 7 weeks of age after weaning. From 7 weeks of age, mice were fed an ND, a 40%, or a 60% high-fat diet (40%HFD, 60%HFD) for 10 weeks.


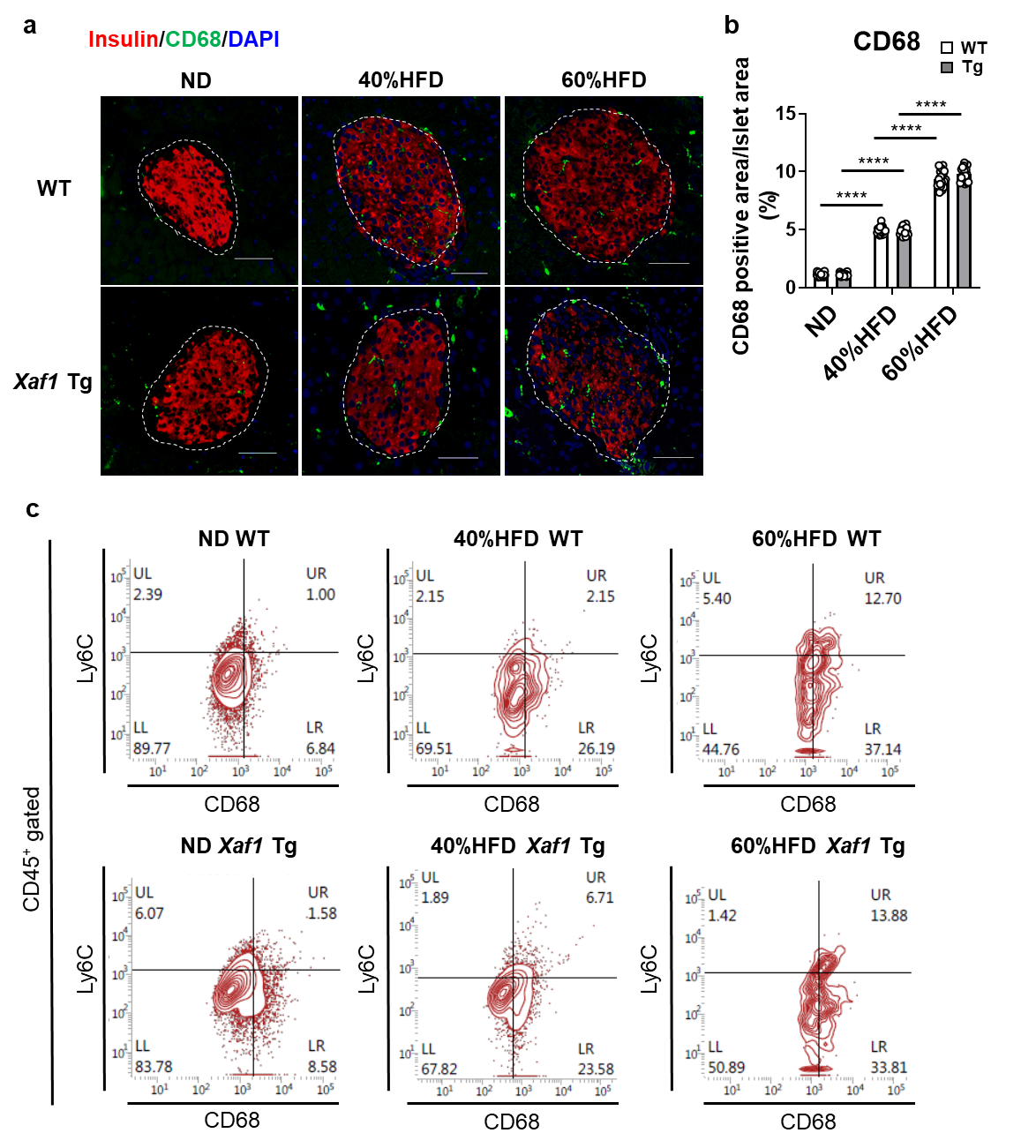
**Supplementary Fig. 5**

**a** Representative images of immunofluorescence staining for CD68 (green) and insulin (red) in pancreatic sections. Areas surrounded by white dotted lines indicates islets. Scale bar = 50 µm, 40 × magnification. **b** Quantification of average CD68^+^ area per islet area. Four animals in each group and 5 random islets per animal were quantified. Data are expressed as mean ± SD. ****p < 0.0001. **c** Flow cytometry analysis of CD68 and Ly6C expression in CD45^+^ islet cells.

Wild-type (WT) and *Xaf1* Tg (Tg) mice were fed with a normal diet (ND) up to 7 weeks of age after weaning. From 7 weeks of age, mice were fed an ND, a 40%, or a 60% high-fat diet (40%HFD, 60%HFD) for 10 weeks.

**Supplementary Fig. 6**

**a**

**
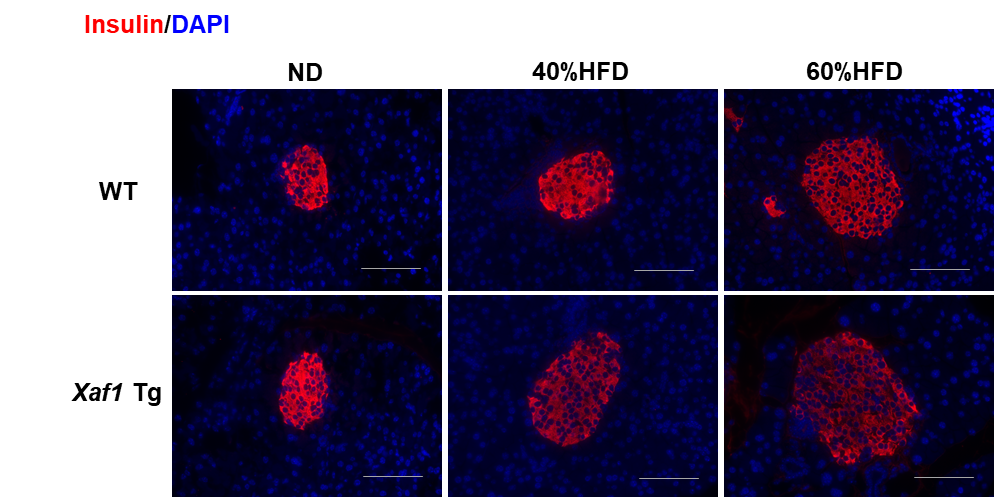
No primary controls**

**
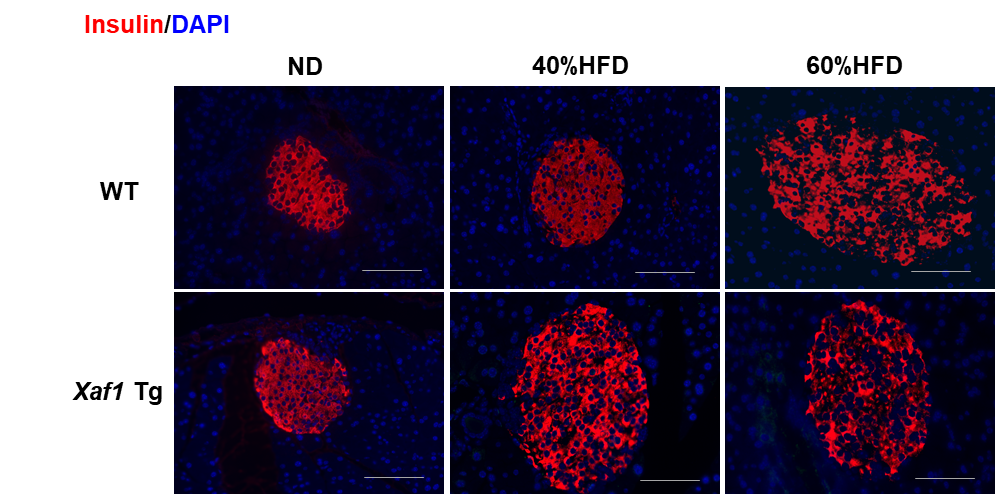
Isotype controls**


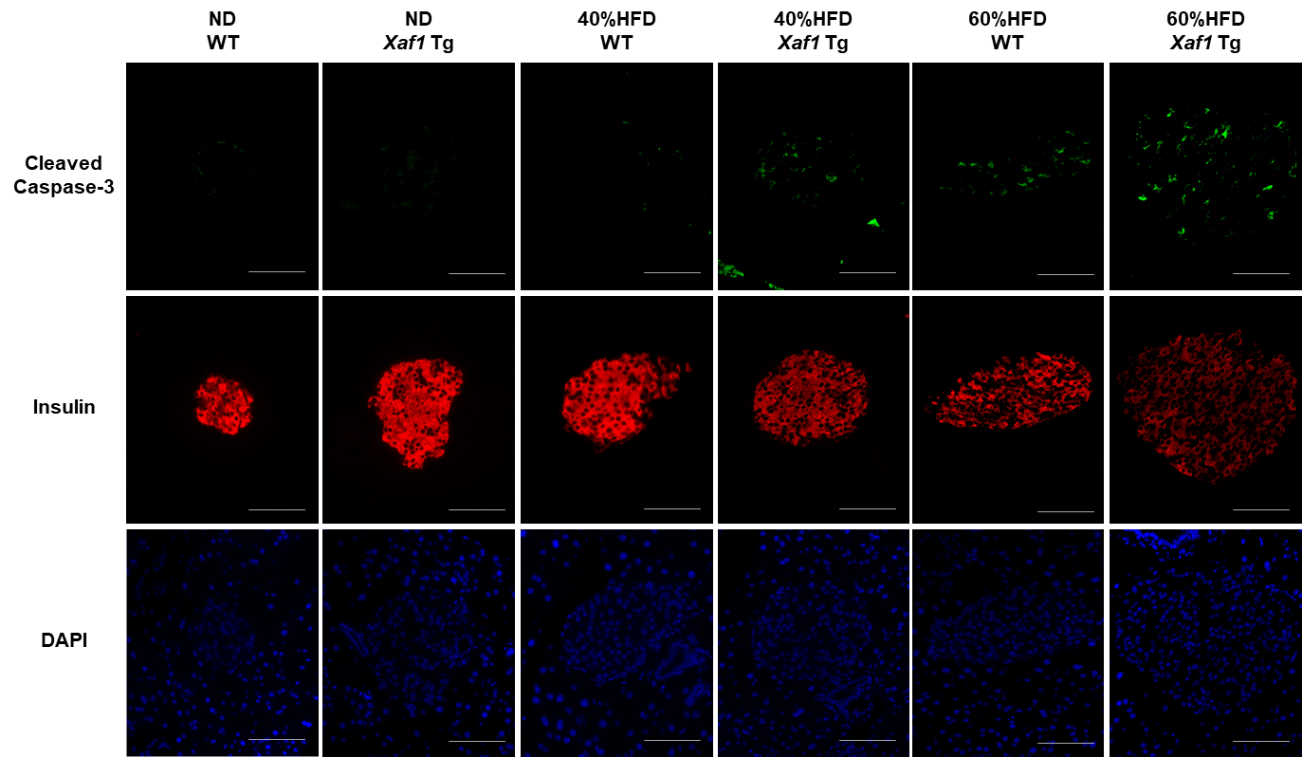
**b**

**a** Representative images of no primary antibody controls and isotype controls of cleaved caspase-3 immunofluorescence in pancreatic sections. Nuclei were stained with DAPI (blue).

No primary controls: The samples were incubated with only mouse anti-insulin antibody at 4°C overnight. After incubation with the primary antibody, the sections were washed and incubated with secondary antibodies for 2 hours in the dark at room temperature.

Isotype controls: The samples were incubated with the isotype control antibody (Rabbit (DA1E) mAb IgG XP® Isotype Control) and mouse anti-insulin antibody at 4°C overnight. After incubation with the primary antibody, the sections were washed and incubated with secondary antibodies for 2 hours in the dark at room temperature.

**b** Representative images of immunofluorescence staining for cleaved caspase-3 (green), insulin (red) in pancreatic sections. Nuclei were stained with DAPI (blue).

Wild-type (WT) and *Xaf1* Tg (Tg) mice were fed with a normal diet (ND) up to 7 weeks of age after weaning. From 7 weeks of age, mice were fed an ND, a 40%, or a 60% high-fat diet (40%HFD, 60%HFD) for 10 weeks. Scale bar = 50 µm, 40 × magnification.

**Supplementary Fig. 7**

**
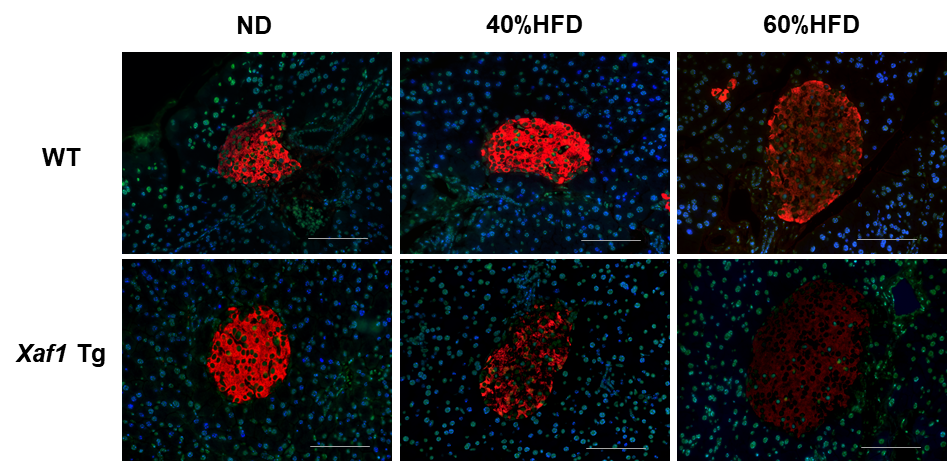
TUNEL positive controls**


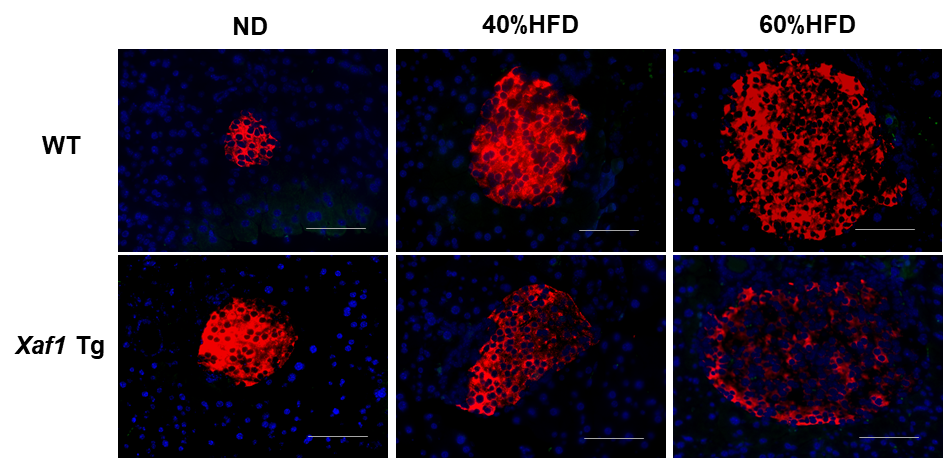
**TUNEL negative controls**

Representative images of TUNEL positive and negative controls in combination with insulin immunofluorescence staining (red) in pancreatic sections. Nuclei were stained with DAPI (blue). Appropriate positive controls were induced DNA strand breaks with recombinant DNase I (Sigma-Aldrich Japan, Tokyo, Japan) treatment before TUNEL labeling. For negative controls, TUNEL staining was performed without TdT enzyme.

Wild-type (WT) and *Xaf1* Tg (Tg) mice were fed with a normal diet (ND) up to 7 weeks of age after weaning. From 7 weeks of age, mice were fed an ND, a 40%, or a 60% high-fat diet (40%HFD, 60%HFD) for 10 weeks. Scale bar = 50 µm, 40 × magnification.

**Reference**

1. Doliba NM, Liu Q, Li C, Chen P, Liu C, Naji A, et al. (2017) Inhibition of cholinergic potentiation of insulin secretion from pancreatic islets by chronic elevation of glucose and fatty acids: Protection by casein kinase 2 inhibitor. Mol Metab 6(10):1240-1253. <https://doi.org/10.1016/j.molmet.2017.07.017>
